# Notices of the Lunatic Asylums of Paris

**Published:** 1845-01

**Authors:** John Conolly

**Affiliations:** Fellow of the Royal College of Physicians, Physician to the Country Lunatic Asylum at Hanwell


### NOTICES OF THE LUNATIC ASYLUMS OF PARIS.

*In a Letter addressed to* John Forbes, m.d. f.r.s.

By John Conolly, M.D.

**Fellow of the Royal College of Physicians, Physician to the County Lunatic Asylum at Hanwell.**

My dear Sir,—You have at different times devoted so much space in your excellent Review to the subject of Insanity, and Asylums for the Insane, that I think you will be interested in an account of the impressions consequent on some visits lately made to the Lunatic Asylums of Paris, institutions again seen by me after the lapse of sixteen years, during which so much has been done in all the civilized countries of Europe to ameliorate the condition of the insane. If the last five years of my life, passed wholly among patients affected with various forms of unsound mind, can be supposed in some degree to have qualified me for observation, and for forming just opinions of what is suitable to them, my remarks may also, I trust, be of possible use at this time, when many new asylums are about to be erected in England, and benevolent persons, in almost every county, are directing their attention to the condition of the pauper lunatic, where no such asylums exist.

Having had the pleasure, in July last, of seeing M. Battelle at Hanwell, who is the director of the civil hospitals (*Hospices civils*) of Paris, and being consequently acquainted with the particular attention paid by him to the construction and government of asylums for the insane, I availed myself of his kind permission to call upon him soon after my arrival in Paris; and I met from him, as indeed from all the public officers of asylums whom I had occasion to wait upon in France, the most cordial and obliging reception. The memory of the agreeable interviews which I enjoyed, in the course of a visit of ten days, with the various doctors and other functionaries of these institutions in and near Paris, would always continue to be dwelt upon by me with gratitude and with pleasure, if even I had not met with still more to admire in the institutions over which the influence of their intelligence, zeal, and philanthropy extends. But I derived so much satisfaction from much that I saw, that I assure you when I turn to the hasty notes made in the wards as I passed through, I could wish to recall hours spent so delightfully, and with so much advantage, and to do fuller justice to the French asylums, by completing as descriptions what I am only able to offer you as sketches.

### THE SALPETRIERE.

The first asylum which I visited was the Salpêtrière, a part of which immense institution is appropriated to insane women; of whom there were 1600. M. Battelle accompanied me; and we found M. Falret, one of the physicians of the asylum, sitting in the schoolroom, a somewhat small but comfortable apartment, in which were collected about 100 of the patients, all perfectly orderly, all neatly dressed, and appearing to take as much pleasure in the occupations of the school as those who witnessed them.

To any one accustomed like myself to the daily observation of the insane, the mere appearance of these patients gave eloquent testimony concerning their general good and kind management. Some were engaged in needlework, which they chose to continue whilst attending to the singing, recitations, and other proceedings of the school. A few only were absorbed in ideas which no change of place can always relieve. None appeared to be in any way troubled or fatigued. All were neatly dressed; their handkerchiefs and caps presenting the variety and some of the singularities always seen when the dress of insane females is not regulated by severe general rules. Above all, almost every one was cheerful, and regarded the attendants, officers, and visitors, without the least indication of suspicion or dislike. A few of the attendants were sitting among them, and by their participation in all that was done, contributed to the general good effect.

As the institution of schools in the Hanwell Asylum has been a favorite object of my ambition, but one in which my hopes have been frustrated, in consequence of their suppression by an authority which I have no power of resisting, it was not without the most singular gratification that I beheld Dr. Falret sitting among his patients, like a father among his children, encouraging them, assisting them, directing them, and promoting all kinds of easy and agreeable intellectual exercises that might diversify the time for the afflicted objects of his care, and, by gentle efforts, lead perhaps, in not a few cases, to the gradual restoration of those powers with the loss of which all is lost that is worth preserving. The tranquillity, the content, the cheerfulness of that little room I shall never forget: and I trust that the hope such a spectacle inspired of being some day aided in a like attempt among the insane of my own country, will yet be realized before my mortal labours are concluded. At least I trust that the directors of other English asylums will not be discouraged from such an attempt. To have abolished all the horrible instruments of restraint from asylums, and to have substituted the more efficient and beneficial restaint of invariable kindness, is a great work, in which many of our English physicians, and directors of asylums, have taken part; but they must not imagine that having done this, they have done all. The perfection of their work remains to be undertaken. The end for which this auspicious liberation is but a means must yet be obtained. The troubled brain has been composed, and the heart of the insane tranquillized ; it now remains to be seen how far the exercise of the intellect can be restored, and the still more valuable empire of well-ordered affections can be regained. Such aspirations are not the mere results of impressions arising in a scene like that witnessed at the Salpêtrière ; they have been strengthened, if not formed, by seeing, day after day, among the poor objects of our care at Hanwell, the successful efforts of a kind schoolmaster and schoolmistress, with the occasional direction of the chaplain, to teach many very suffering patients to read, to write, to draw, to sing ; efforts rewarded by the obvious happiness produced by such occupations, and the perceptible improvement of the mental faculties of some of those engaged in them. When these efforts were no longer permitted, it was among my consolations that Hanwell was not the only asylum in which they had been commenced. The previous labours of MM. Ferrus, Leuret, Voisin, and Falret, were not unknown to me ; and to witness the happy continuance of those efforts would alone have repaid me for the journey to France.

The patients at the Salpêtrière have the advantage of a library, and several of them have read parts of the excellent books allowed for their perusal with so much attention as to be able, when requested, to recite them for the amusement of the other patients. Three or four of the women, in the schoolroom, were called upon in succession by Dr. Falret to do this. Each immediately stood up with much cheerfulness, and distinctly and pleasingly recited a short story or poem. This was done with great correctness; and it seemed as if the patients knew the whole of some long poems, which they went on reciting until stopped, when they sat down with an equal air of content. During the recitation many of the other patients appeared to be attentive hearers. Several of the patients were then invited to join in singing something; and they sung several verses, and in parts, very correctly and agreeably, and apparently without any sane leader. Afterward an Italian patient sung a beautiful air with considerable skill, to the evident satisfaction of her companions. I saw various specimens of their writing, which were excellent: it was, indeed, with regret that I left this part of the establishment, where, by means of innocent and improving recreations, the patients pass a portion of each day in tranquillity, and, it may even be said, in happiness; and it will be long before I lose the wish to see those cheerful grateful groups again. The school at the Salpêtrière is only a part of what has been done, and what I shall have to describe to you, for the instruction of the insane in Paris.

This place of careful instruction was but a preface to the whole of the establishment, which I found to be remarkable for its cleanliness, order, and tranquillity. I saw no patients walking about in strait-waistcoats, or muffs, or leg-locks, or handcuffs; and I heard no sounds of raving or fury. At a former visit, in 1828, the restraints and their constant accompaniments had not yet disappeared. In a very recent visit made to them by my son, traces of them were still visible, and attracted his attention by reminding him of our difficulties at Hanwell in 1839. But on the day of my recent visit there was, in fact, not one patient in restraint in the whole asylum. A few were in cells of seclusion, which are detached from the building occupied by the tranquil and industrious. It is true that the French physicians hesitate to admit the principle of the *total* abolition of mechanical restraints, but they employ them rarely, and with so much reluctance as to give every assurance that the period of the entire disappearance of the camisole is not far remote. To all the violent modes of restraint, however ingenious, they have, I think, always been opposed. M. Mitivié showed me an apparatus of iron and leather which he had some years since procured, I am sorry to say from England, in consequence of the very high praise bestowed upon its convenience, comfort, and even elegance; it is a most oppressive belt for the body, with chains and fetters for the hands; and it is gratifying to know that its repulsive appearance deterred M. Mitivié from ever using it. The case of Morris, immortalized in the plate attached to the work of Esquirol, is yet referred to with astonishment, and almost with incredulity. The whole spirit of the treatment of the insane in the French asylums appears to be mild, gentle, and humane; and the seeming exceptions to it, which I shall have to notice in speaking of other institutions, are to be ascribed to the same misapprehension concerning the indispensable employment of restraints in certain cases which yet impedes the progress of their abolition in some of our own asylums.

There has been a reciprocation of benefits between the two countries, which it is not unpleasant to reflect upon. Before the assigning of a part of the Salpêtrière to the reception of lunatics, and when these unhappy creatures were kept in narrow dormitories containing four rows of beds, each bed containing three or four restless patients, Tenon visited the English asylums, and he returned to Paris confirmed in his views of reforming this state of things. But even this good man conceived it necessary to project long corridors, with cells and chains on each side. Within ten years from that period, in 1792, Pinel liberated fifty lunatics from chains, which many of them had worn for years, and applied himself to the general amelioration of the condition of the insane with a zeal and success which will make his name remembered with honour for ever. About the same time the Quaker's Retreat was established at York, an institution in which humane principles wholly predominated, and to which all French authorities refer with respect. Early in the present century M. Esquirol began to second and to extend the beneficial labours of his friend and master Pinel. The frightful mismanagement of the York Asylum, in the very neighbourhood of the Retreat,—screened from exposure by all the authority of its committee, honourable men, slow to suspect and studiously deceived, somewhat ignorant also and jealous of authority—until the misdeeds and inhumanity of their officers could be concealed no longer; and the subsequent revelations consequent upon a Parliamentary investigation into the state of other institutions, led, it is well known, to a general improvement of the state of English asylums; in many of which no instrument of mechanical restraint is now to be found. If the French physicians yet hesitate to adopt this last and not least improvement, let us not forget that in their attempts to instruct the insane they have gone before us; and that, by the generous aid of the authorities under whom they act, they are setting in this respect an example most worthy to be followed by all other nations.

It is with feelings of the deepest mortification that I acknowledge how fatal to the speedy abolition of restraint in France must be the Report of the Metropolitan Commissioners of Lunacy, now translated by M. Battelle. The single example quoted from the male side of the Hanwell Asylum was a mere accident; but I am more grieved than surprised to find examples cited from the female side to show its inconveniences. The causes of this are involved in the general question of the necessity of having some fixed regulations for the government of asylums, which I hope will eventually occupy the attention of the governing bodies of those institutions, or of the legislature. In this respect I am inclined to think that both the continental and the Irish asylums are better managed than the asylums of England; and that there is consequently a prospect of more permanent and steady improvement in them.

The greater number of the buildings assigned to insane persons in the immense hospital of La Salpêtrière (where also the aged and infirm find an asylum or almshouse) consist of one story only, and this is divided into a large dormitory and a moderate sized day-room. There are several small tables in the day-rooms, covered with neat-looking oil-cloth. As the space between the beds in the dormitories is but an indifferent substitute for the ordinary galleries seen in asylums, it would be well if the day-rooms were larger. At present there is a want of space for exercise within doors. The dormitories are, however, wide and lofty, and generally contain thirty or forty beds: one of them contained 150 beds, and is too crowded. Their state of cleanliness is perfect; and although possessing only the ordinary modes of ventilation by the doors and windows, no bad smell was anywhere perceptible. Some of the floors are of polished oak, and some of brick. The beds are on a raised platform of oak, and those for the dirty patients have a large stone slab underneath them, and an oak flooring between each. At the side of each bed there is a chair; generally speaking the beds are superior to those in any English asylums. The beds for the sick are provided with white curtains. An air of perfect comfort prevails throughout; and a tranquillity perhaps even greater than desirable. Our patients at Hanwell are so lively and communicative, that I have been surprised by the general silence of the French asylums, and am unable to account for it. The difficulty is increased by a consideration of the habits of social life among the French, on which the directors of their asylums found their strongest argument for large dormitories and against single sleeping rooms.*

Constant experience of the inconveniences attending even a small number of dormitories, for twelve or eighteen patients, at Hanwell, has long caused me invariably to warn architects against them, and to recommend that although a certain number of smaller dormitories may be desirable, chiefly for the suicidal or the timid, each containing four or five beds, there should be single sleeping rooms in every asylum, in the proportion of at least two thirds to the whole number of patients to be accommodated. The violent must have single rooms; the mischievous must be placed in them for security, and the helpless for protection, unless the aggregate of restraint in an asylum is allowed to become excessive; the dirty generally require them; and of the tranquil a great many are constantly dissatisfied if obliged to sleep in rooms with others, and complain that they cannot sleep, and that they are annoyed by the language of the other patients. In a large dormitory one restless patient disturbs all the rest. The most tranquil patients, and those who are convalescent, are always petitioners for a single room: it becomes a kind of house to them; they attend to it themselves; they sit and write or read in it; they decorate it, and are made comparatively happy by its possession. If dispossessed of it, and forced into a room with others, they become fretful and discontented.

The character of French patients appears to be different. Nearly all the physicians, as well as M. Battelle, and M. Ferrus, the director-general of the asylums of France, assured me that the establishment of large dormitories had produced great content and tranquillity in the asylums; and they ascribed this to the general habits of social life among the French people. Consequently single rooms are only given to the violent, and as an indulgence to some of the convalescents; an indulgence which it is therefore presumable that they really do appreciate.

As large dormitories have been recently recommended by the Commissioners of Lunacy, chiefly on the plea of economy, a plea which is always popular, and generally delusive in matters of building, I fear they may be adopted in some of the new county asylums in England; but I believe the general testimony of superintendents in England would be strongly against them. On the testimony or example of private houses, licensed for the reception of pauper patients, I place very little reliance. Economy is the vital principle of such establishments; and if their patients sleep in large dormitories without doing much mischief, it is because the restless are fastened to their beds, and the disturbance they occasion to others is disregarded. In large dormitories there must be attendants at night, which I think objectionable, as dis-

* In consequence of the crowded state of the Parisian asylums, several patients had been sent from them into the provinces, and among them some of the most refractory. M. Trélat's account of the removal of these patients, 400 in number, is singularly interesting and instructive. (Annales Médico-Psycologiques, Sept. and Nov. 1844.)

qualifying them in a great degree for active duty by day. They cannot enjoy needful rest; and how inconceivably harassing their daily duties are can only be known by those who are accustomed to spend a considerable part of the day in lunatic wards. Among other considerations of their general condition, too little attended to, it is most essential to their bodily and mental efficiency that their nights should be undisturbed, except when their turn of guard or night duty comes, which ought not to be oftener than once in ten or twelve nights.

This is one of many points relative to lunatic asylums on which erroneous opinions are very likely to be indulged by all except the very few who are witnesses of what takes place in the hours of night as well as in the hours of day, in such institutions. A large dormitory looks neat and comfortable by day; the beds are in nice order, the floor is clean, the air is tolerably pure, and the room is quietness itself; but a dormitory full of patients who have been a few hours in bed is a very different place; the atmosphere is heated and oppressive, and every excitable patient is restless. Even in the daytime the air in the dormitories is generally less pure than in the single bedrooms. The entreaties of the more rational of the patients to be delivered from this purgatory, to which they generally give a warmer name, are such as very strongly express their sense of its discomforts. These are evils which ought not to be incurred merely to avoid expense; and there is no reason why many single bedrooms should have gloomier windows than those of dormitories. I greatly regret, therefore, that the commissioners, whose opinions will justly have great weight and authority, should have recommended large dormitories on grounds which appear to me to be so entirely unsatisfactory. (See Report of the Commissioners, 1844, pp. 12, 13, and 21.)

The French have naturally enough fallen into the error of supposing that the seclusion of patients at Hanwell, is a long imprisonment in dark and solitary rooms; as represented by the Commissioners. In the Hanwell Reports, the practice of seclusion is fully explained to be a simple exclusion of all irritations for a limited time, and of which the effects are most carefully watched, and known to be most salutary. The opinion of the commissioners was not the result of observation.

The buildings at the Salpêtrière, and in the other French asylums which I have visited, have less regularity of form than the more recent structures of a like kind in England; but they possess some advantages for the separation and classification of patients in the detached parts of which they consist; and they are free from the basements and upper stories that disfigure many of our asylums, and render them so difficult of ventilation, and even of proper inspection. Most of the buildings consist only of apartments on the ground floor; and they are separated from other buildings for other classes of patients, by large airing courts, in which there are trees and grass, and flowers; these courts, like those of Hanwell, having undergone much recent improvement.

But the inconvenience of several separate buildings, consisting only of one floor, or of two floors, is so great as regards the supply of provisions, as well as for the general purposes of supervision and attendance, as, I think, to form an insuperable objection to such a plan; although it has had the support of great authority; and, upon the whole, I am convinced that a straight continuous line of main building, extending from a central house and offices, is the best as well as simplest form for an asylum. This cannot well be prolonged further than for the accommodation of about 240 patients; in eight wards, containing about thirty patients each; which wards are best suited to the tranquil and clean, and moderately tranquil and orderly. The sleeping-rooms ought to be all on one side of the galleries of these wards, the galleries having large windows (in our climate,) toward the south. Wings at right angles with the main building, will provide for 120 more, including the more turbulent and untidy in four wards, each containing twenty-five patients, the galleries looking west and east. Ends of a T shape may be contrived to accommodate forty more; but it is not desirable to enlarge the wards for this description of patients, who are best managed when subdivided into smaller numbers.

Up to this point no great difficulty presents itself. Light, air, warmth, provisions, stores are easily distributed through the building, and each ward can have a separate airing court without destroying the classification secured by the division of wards; a point often forgotten; patients of various character as regards their malady being mixed in the airing-courts, and the windows of quiet wards overlooking refractory airing courts. This, therefore, should be considered the limit of the extent of an asylum. Its *maximum* should not exceed 400 beds. Its size, and the probable amount of remuneration will then be sufficient to secure able officers; whilst it may be conveniently and sufficiently superintended by one physician, with a dispensing assistant, and thus a uniform plan of management may be ensured. But beyond this extent, difficulties arise and accumulate. The impossibility of extending the building without impeding the access of air and light, or producing some unthought-of disadvantage, perplexes architects; and they are driven to add third stories, or seduced into excavating gloomy basements; so as to crowd a greater number of patients into a given space than ought to be collected in one building. It is then that two or three systems begin to prevail in the same house. Its form of government becomes insecure: and considerations of health as well as of convenience are often sacrificed to that of economy, or to pressing necessity. For these and other reasons, although I think large asylums in every way preferable to small ones, I am of opinion that the size should never exceed that of a building capable of containing 400 beds.

The quantity of land originally secured for a lunatic asylum is generally found to be insufficient for all the purposes of exercise and occupation. A comparison of the quantity found desirable in the best asylums with that attached to asylums in general seems to establish it as a rule, that where both male and female patients are admitted, it should be nearly in the proportion of ten acres to every hundred patients. In the Salpêtrière it is much below this; but all the patients are women, and employment out of doors is not required.

In the interior arrangements of the French asylums the dormitories are generally so situated as to open by a wide door into a commodious day room, in which some of the patients work, and all take their meals. The greater number of the patients are now, however, in separate work-rooms during the day and evening, except when out of doors, and in those rooms they have constant superintendence. In our asylums, where some of the patients are in the galleries, occupied, or merely walking about during the day, and others are in the day-rooms, which do not open directly out of the galleries, the superintendence is very inefficient. The difference is remarkably observable at Hanwell. In the refractory wards there are no day-rooms, and when the door of the ward is opened the visitor immediately sees the attendants together with the patients; whereas, in the quieter wards, the visitor has often to walk through a long gallery, and among numerous patients, before arriving at the day-room, where the attendants are out of sight and hearing of what passes in the gallery. For this reason I have for some time advised the omission of day-rooms in the plans of asylums, and the substitution of wider galleries, with deep bays or recesses, which plan is, I believe, adopted in the Derby Asylum. But I think it also highly desirable that there should be several detached rooms for employment, and some for recreation, all provided with proper attendants.

In the modes of warming and ventilating the French asylums there is nothing worthy of observation. Stoves are in general use, and the dormitories have large windows on both sides. The detached cells for the refractory are not warmed. There are no questions more difficult than those which relate to the best modes of obtaining warmth and fresh air. Warming by steam is not a convenient way of preserving an equal temperature for many hours. Hot water is less open to that objection, but requires many repetitions of the apparatus. The more elaborate method of introducing warm fresh air, and removing foul air from the galleries and bedrooms, as in the Pentonville and Derby prisons, are perhaps the best; but every plan of ventilation should permit the frequent access of fresh air by windows; and whatever mode of warming is adopted in an asylum there ought to be an open fireplace in every ward.

The number and the comfort of the separate rooms for work, or reading, or recreation, at the Salpêtrière are among the great advantages possessed by this asylum. In more than one there is an excellent piano, and patients who are fond of music are permitted to play and sing there almost whenever they choose. These rooms are of various dimensions; some very large. In one large work-room there are 150 patients, some at needlework, and many employed in making, under the direction of a patient, very comfortable shoes of coloured worsted, on a last. In other rooms I saw many patients collected together for employment, or sitting down to dinner, who were formerly considered so refractory as to make such social assembling impossible. One hundred and forty-three chronic cases were in one such apartment at dinner; and in another eighty-four, of whom the greater number were advanced far into dementia, and many of whom were to be seen, only three or four years ago, lying on the floor, and presenting the most lamentable spectacle. The ameliorations in this part of the asylum are all of very recent date, and very striking. Rooms have been built for these poor people, and their condition raised at once from abject wretchedness to comfort. The number of those capable of being usefully employed has been found great beyond expectation, even among the least intelligent, the most inactive, and the least orderly of the patients; and idleness has become the exception. In the accomplishment of these great ameliorations I must not pass over the name of M. Trélat, whom I regret not to have seen, but whose exertions have been great and rewarded by surprising improvement in many of the patients. When beneficial changes are made, the pains it costs to make them are too much forgotten. When wards are reduced to order, and cleanliness is established, the meritorious attendants who have effected this are liable to dismissal to make room for new ones; and when one sees a long table, surrounded by a crowd of lunatics, dining in comfort, and using knives and forks, one may forget that here formerly each wretched patient devoured his food out of a dirty wooden bowl, crouching apart, like an animal of prey. Much that M. Trélat hopes to do yet remains to be accomplished; but even now the patients work together, dine together, have good beds, and are cheerful and tranquil; whereas they were formerly turbulent, slept on straw, and realized in their appearance the worst features of a madhouse.

Nothing does more honour to an asylum than the care and protection it extends to the imbecile and helpless. These unhappy beings may be neglected to a great degree with impunity; and that in the old asylums they were grievously so, is too well known. Among the objects which gratify me in every visit to Hanwell, none is more entirely satisfactory than the extreme attention paid to the most helpless of the patients, the imbecile, the idiotic, the paralysed, and all who have fallen into the utmost weakness of mind and body; a state in which they possess no interest for the ordinary spectator, whom they neither alarm by fury nor amaze by eccentricity. Unlike the less heavily afflicted, they can neither appeal to the philanthropy of the visitor nor to the authority of inspectors; and they would be lost if no compassion were excited by their very wretchedness and squalor, which, however, long pleaded silently and in vain. Among these are not a few whom the physician has traced through successive stages of mental and bodily decay, from the first storm of unreason to the last wreck of sense and intelligence, and who, he knows, can have no friend on this side of the grave if he ceases to be such. It is these abject creatures who have been rescued by the active benevolence prevailing in asylums from a state in which it was thought impossible to produce them to decent view. Many a wretch heretofore doomed to lie in hopeless neglect is now daily dressed in clean and warm clothing, and brought out of his bed to sit by the fire or to breathe the fresh and invigorating air. A feeble smile of recognition still passes over the features of these poor declining patients, and not a few of them utter words expressive of their content. They are reduced to the condition of children, and they are treated as children, fed as children, kept clean like children, put into bed like children; they are only not punished like children; but are guarded by night and by day from danger, violence, or neglect, until their poor remains of life can be husbanded no longer.

But for the quality of mercy, in these later times only fallen into their secluded cells, still, among these doomed creatures, in solitude and darkness, and foul smells, the old instruments of restraint would be triumphantly applied as substitutes for all trouble; and wretches still would linger and toss in straw and filth for months and years, through life to death, uncared for and unknown. Those days are past; but their heavy shadow still remains, and obscures humanity still in a kind of defiance of public inspection, and the public exposure of official Reports of the Commissioners of Lunacy. The persevering enmity, too, which has been steadily directed against the reformers of these things, has assumed, year after year, and still assumes so many ingenious shapes, that the friends of the insane must not permit themselves to be lulled into security, or believe that a gradual return to such things is quite beyond possibility. The old arguments against non-restraint, founded on a supposed necessity for other forms of cruelty in the place of restraints, or of a great destruction of clothes and windows, are still continually brought forward by those who first committed themselves by a demonstration of affection for fetters of iron and ligatures and muffs of leather; and the same insinuations are repeated of the insincerity of those who abjure the use of such mechanical methods in the treatment of infirmities and griefs of the mind. But with all this, humane principles proceed unchecked, and are silently working their blessed way into all the lunatic asylums of the world. A few of the old institutions of England, very few, may yet seem ambitious curiously to illustrate means of coercion becoming unknown elsewhere; but throughout Europe and America and Asia, such things will soon be rare. Already has an able and humane physician gone from Hanwell, by the express wish of the local government of Ceylon, to effect improvements in the condition of the lunatics of that distant colony; and, in a few years more, the traveller may have to record ameliorations even in the woful asylums of Cairo and Constantinople.*

When I contemplate this, the worst class of incurables, I always think with concern of the disposition apparent in some quarters to make a formal separation, in distinct asylums, of the *curable* and *incurable.* Such propositions are abstractedly pleasing; but the effect ought to be well considered. In the case of many incurables, the manners and habits during a great part of every year are superior to those of the curable, and, therefore, not detrimental to curable companions. The separation and transmission to an asylum for incurables would be felt by them as a sentence of eternal imprisonment. As it is, hope, the only blessing left, never dies within them; and some of them recover, after years of affliction, and beyond any ordinary expectation. At once to pronounce many cases incurable which seem so would be to commit many errors; and the asylum for those pronounced curable would always be half filled with cases really not to be cured. The only class, therefore, of the incurable who could safely be severed from the rest of the patients, as mentally dead, would be these poor imbecile, helpless creatures, fallen into fatuity and dementia: and there is much reason to fear that by separating them from a curative asylum they would lose all the benefits which alleviate their unhappy lot. It is quite unnecessary to mix them in wards with more intelligent patients; but it is surely humane to keep them under sufficiently active medical direction in an asylum where it is admitted as a principle, that if few cases admit of cure, every case admits of improvement. What I shall presently describe will show you that more can be done, even as regards the most idiotic patients, than their aspect gives any promise of.

The number of the epileptic and idiotic in the Salpêtrière is considerable, not less I believe than 400. The epileptic, of course, vary greatly in degrees of intelligence, and some of them appeared to be too sensible to be properly placed with the

* I admit the serious importance of one objection made to the non-restraint system, as practised in some asylums; namely, that whilst everything humane is professed, the attendants may practise an habitual severity and be guilty of secret cruelty. This is but too possible; although I believe nothing so rapidly deprives the attendants of all humanity, or so effectually and certainly brutalizes them as the habit of resorting to restraints. The obvious remedy for this suspected cruelty is never to overlook a known instance of cruelty or of wilful neglect. There is no other protection for the patients, and an asylum is ill governed where the physician cannot afford this protection to his patients.

In a printed letter addressed to Lord Ashley, dated 1844, bearing no name of author or printer, such very grave charges are made against those who have adopted the humane system as demand strict investigation. The expressions and sentiments in the letter, as clearly demonstrate its origin as if the matron whose office is subscribed to it had affixed her name to it. But with all the deductions thus rendered necessary, there are some things set forth in the letter which at least show the desirableness of a searching inquiry into the actual state and government of County asylums, and of some uniform laws being devised for the protection of the patients and also of the officers.

idiots and perfectly imbecile patients whose malady was more advanced. A subdivision of the epileptics is desirable in all asylums, as many of them are quite rational for long intervals between the attacks of a disorder which finally destroys the mental faculties. I did not observe any particular precautions against injury from falls, from which the epileptic suffer so frequently. The floors of the large apartments in which they are placed are of wood; but it does not seem that any kind of cap is considered useful; and this quite accords with my own experience: as there are few of these patients who fall in such a manner as to render it possible for the head to be protected by any kind of cap, and fewer still who will long wear a cap if put on for that purpose. It is a singular feature of this malady, when combined with insanity, that in many cases the patient seems to have no consciousness or memory of the successive attacks, and will report himself quite free from them, although, perhaps, only recovering from convulsion and stupor.

The notion so obstinately cherished by the lovers of restraint, that it is necessary to fasten every epileptic to his bed at night, in order to prevent turning on the face and suffocation, does not appear to have found its way into the minds of the French physicians, and the pernicious practice founded upon it is unknown.

As many as eighty idiots and epileptics were in one day-room at the Salpêtriére, and the confusion was less than one would have expected. I should be inclined to waive my objection to large dormitories in the case of the epileptic, who are frequently subject to fits in the night, which are sometimes fatal. Several of them are, however, so liable to accessions of sudden and violent mania as to require separate sleeping-rooms; and sudden deaths will occur among the epileptic by night and by day, without the possibility of relief or assistance from those nearest to the patient at the time. There is, perhaps, no class of the insane whose character is more injured by restraint than the epileptic; their attacks of excitement being so frequent, that if always met by restraint, the patient passes, but a small portion of his time in perfect liberty. The cheerful appearance of these patients in the crowded apartments at the Salpêtrière, and the perfect order and tranquillity observable in the male ward of Hanwell entirely filled with epileptics, and which I remember one of the most turbulent and dangerous wards in the asylum, sufficiently declare the advantage of non-restraint in this class of cases; in which, at the same time, all the ordinary and extraordinary difficulties of maniacal violence require to be provided against.

Among the difficulties attendant on the management of an asylum, the care of those unhappy patients whose malady has incapacitated them for attending to personal cleanliness presents not a few. Under the old system, looked back to with such fond regret, restraints were the only resource. The patients were left to wallow in dirty straw, horrible to view and smell, but secure. In the Salpêtrière there is nothing in the atmosphere to notify to the visitor that he is in wards devoted to this class of patients. The wards for these patients contain about twenty-four; they sleep on thick beds of straw, and all ordinary inconveniences are avoided by scrupulous attention to its cleanliness. But I neither think this kind of bed, nor the perforated bedding of the Bicêtre, protected by a waterproof covering, nor the beds of sea-weed also used there, so unobjectionable as our simple *frame-beds* at Hanwell; which consist of a piece of strong canvass fixed on a light frame, fitting on to each bedstead, and easily removeable; the bedstead or crib being lined at the bottom with lead, sloping to a central perforation. These were adopted after long trial at Gloucester and at Nottingham, and under their use we have had none of the terrible cases of ulceration of the back, which were both common and fatal, when troublesome or helpless patients were fastened down, or were kept long lying on straw. Most of the contrivances for the beds of dirty patients presuppose this night-restraint to an extent which does not allow the patient to turn, or even to lie comfortably on either side.

Several paralytic patients are placed in these wards, chiefly presenting the form now so generally recognized in lunatic asylums, and so little known anywhere else, to which the name of General Paralysis, or Paralysis of the Insane, has been given. In this unhappy combination of paralysis and insanity, so difficult to account for, so much more common in men than in women, and in the temperate than in warmer climates, the speech becomes early and peculiarly affected, and by degrees the patient reaches the utmost stage of helplessness. My own observation leads me to pronounce all such cases absolutely hopeless, and to say that the slightest tremor of the lips, or affection of the tongue peculiar to it, is of fatal omen. But it is only right to say that some physicians are more sanguine, and among them M. Falret, who is of opinion that heroic counter-irritation of the spine checks the progress of the malady. Its progress is certainly much retarded by care and proper nourishment, and the patients grow fat, and are distinguished by a remarkable and most characteristic exhilaration of mind. I first saw cases of this kind at Charenton in 1828, when they were pointed out to me by M. Calmeil, who first gave a full and distinct account of this singular malady. We have always numerous examples of it in the Male Infirmary at Hanwell. The old restraint-chairs were often occupied by these unfortunate patients; but I did not see one of these in use in Paris.

A part of the building at the Salpêtrière consists of rows of single cells for violent patients, quite detached from other wards, and opening on a wide airing-court. In these everything is done with a view to security, but I saw no apparatus of chains or restraints of any kind. Very few of the cells were occupied; and, as I have before said, there was not a patient in a strait-waistcoat in the whole asylum. The tranquillity of what might be called the refractory wards was perfect; and the general sense of safety felt by the attendants and officers may be deduced from the circumstance of all the patients in the asylum (with occasional exceptions) being allowed to use an ordinary shaped knife and fork at dinner.

The means of administering baths of various kinds, for the repression of excitement, for the relief of oppression, or for mere comfort and cleanliness, is a very important part of the arrangement of an asylum, and one to which the French have paid great attention. The baths are many in number, shallow, and somewhat coffin-shaped, and generally in one large apartment. Several patients are to be seen almost at all hours of the day in the baths, and a lid is affixed to each bath, which secures the patient in it; of course leaving his head out. The warm or tepid bath is thus applied for hours, chiefly as a sedative. Sometimes a large cap-shaped sponge, dipped in cold water, is applied to the head at the same time; and sometimes pails of cold water are thrown over the head. The douche is occasionally used; but I saw no shower-bath. The patients seemed much at their ease in the baths, and some of them occasionally slept. I have myself long discontinued the use of the douche, finding that all its advantages are obtained from the use of the shower-bath, without its inconveniences; the patient being benefited more, and suffering less. On the advantages of the tepid and warm baths it is unnecessary to make any remarks, and I believe the prolonged use of them is sometimes peculiarly serviceable. The French physicians occasionally use the cold affusion alone; but the *bath of surprise* is abolished. Perhaps I ought to explain that this ingenious application of the bath consisted in suddenly throwing a patient, bound hand and foot, into a large cold bath, and repeatedly forcing his head beneath the surface. Many patients have described this to me, and young women have told me that they were conducted to this bath of fear and torture by male attendants. This was one of the ordinary accompaniments of the restraint-system. Can you wonder that I dread the danger of its being restored to favour?

Although my visit to the Salpêtrière was so long that it would have tired out any less kind guide than M. Battelle, I left it with great reluctance; and having the pleasure of afterward meeting the Abbé Christophe at M. Falret's, I was glad to have his sanction for attending with him the next day, Sunday, in his visit to the patients. On going into the large church of the Salpêtrière, at eleven o'clock, I found it filled with the numerous pensioners or alms-women of the institution, excepting about two of the portions of the church radiating from the central altar. Notwithstanding all my familiarity with insane people I at first experienced some difficulty in ascertaining in which of the radiations the patients were seated, so perfectly decorous were they all. At length I recognized the Italian patient, and then others, and placed myself near them, still often doubting whether all those around me were really insane or not; so quiet were they, and so apparently attentive and devout. I stood by them as they moved away at the end of the service, and received many kind nods and smiles of recognition ; but there were very few who exhibited much eccentricity of manner. On passing out of the church they walked, four and four, to their own part of the establishment, with the most perfect order and decorum, and their comfortable dress and general appearance were highly gratifying.

As I differ in opinion from the admirers of restraints on almost every point of treatment, so I especially differ from them in my estimate of the consolation and advantage of religious instruction or conversation by means of a chaplain, which they naturally enough depreciate. The truth is, that restraint vitiates everything, neutralizes all moral treatment, and reflects disgrace and even ridicule on attempts of any higher kind. It is not so where the patients are treated with uniform kindness—I would say like children ; but I ought rather to say like human beings. At the Salpêtrière, as at Hanwell, I witnessed the affecting spectacle of a lunatic congregation listening to a sermon. They adjourned from the church to the school-room, where the Abbé addressed them in a discourse which lasted about twenty minutes. Everything was done with great simplicity. The patients sat on the benches, the female attendants being scattered among them. The preacher sat at the desk usually occupied by the physician, and I was allowed a chair by his side. A few verses of a hymn were sung by the congregation, accompanied on the piano by a patient. The sermon was very plain and clear, and full of kind and hopeful observations. Most of the patients seemed attentive to it : one or two wept, and others endeavoured to console them. One patient alone became restless, said she heard a voice calling to her, and walked quietly out of the chapel. At the conclusion of the service I took a kind of brief general leave of the patients, strengthened, I hope, in every good determination to exert myself for the insane ; more regardless, if possible, than I have been, of personal consequences. We had scarcely left the chapel when a general clapping of hands and shouting took place, and the head nurse followed us to say that it was occasioned by some observation of hers concerning the notice I had taken of the patients. I have often said that lunatics require the most scrupulous demeanour towards them ; they do not like to be treated as if they were childish, and they scrutinize expressions of pity with a strong suspicion of being insulted ; but to friendly greetings and recognition they are very sensible ; and I am not ashamed to confess that long habit has attached me to their unsophisticated manners, of which, at least, flattery forms no part, and that the occasional indications of their feelings and their gratitude are among the things that have bound me so closely to the cause of their welfare.

MM. Falret, Mitivié, Baillarger, and Trélat, all men of eminence, and whose only rivalry appears to be that of introducing improvements, are the physicians who attend to the insane at the Salpêtrière. Each has his distinct division of the asylum. There is a director, who is not medical ; and he is assisted by a steward. In the French asylums there is no matron. There is a chief nurse in each division, acting entirely under the direction of the physician ; and there are female superintendents of the laundry, work-rooms, &c. In each ward there is a nurse and an under nurse ; they wear very neat dark dresses, and caps distinctive of their rank. The head nurse appeared to be an active, cheerful, kind person ; and she receives 360 francs a year, about fifteen pounds English. The under-nurses are paid 120 francs. The greater number of them are young and of respectable appearance. The nominal proportion of attendants to patients is one to ten : but as several of them have miscellaneous duties, the actual superintendence of the patients devolves on a smaller number, probably on one for every fifteen patients, which is not very different from the proportion existing at Hanwell.

No one has derived more advantage in treating the insane than I have done from the co-operation of a matron ; and it is to be feared that where there is no such officer many consolatory offices towards the patients must go unperformed. But the office demands a union of qualities rarely found in woman : great courage, great patience, great temper, great zeal, great humanity, and a willingness to act as an auxiliary to the medical officers. None of these qualities are common ; but the last is the most difficult to be met with, and its absence is the cause of great evil in many asylums and hospitals in England, embarrassing the physician, and neutralizing all his efforts.

In accordance with the general spirit of philanthropy pervading this noble institution, a kind protection is afforded to those who leave it when recovered. It may seem strange to those who witness the very frequent petitions for liberation addressed to visitors in the wards of an asylum to be told that there are not a few patients who quit it with regret and with tears. The dreadful aspect of poverty appals them, and they tremble to leave the threshold where they have been treated with humanity, and have known no want. Yet to keep these destitute persons in the asylum is impracticable; and it becomes, at length, imperative to turn them out again into a world in which it is but too probable they will starve, or, less miserable, become insane again, and qualified for a shelter and refuge elsewhere denied to them. For the relief of these patients the fund called the Adelaide Fund (from its royal and benevolent founder) was several years since established at Hanwell. Still more important relief, however, is given to those discharged from the Salpêtrière. An establishment exists which is intermediate between the asylum and society, affording a temporary home, suitable occupation, medical and moral superintendence, and, after a time, opportunities of resuming the labours and duties of ordinary life. Even the children of these patients receive the care and training which they often so peculiarly demand.

Amidst all the overflowing charity of England, no such refuge exists. I trust, my dear sir, that after being already associated in not a few useful designs, we are neither of us too old to hope that yet before we die we may assist in promoting an establishment so full of merciful influences; and also see a model asylum instituted for those most afflicted persons who, belonging to the educated portion of the middle classes, and depending on the continued exercise of their talents and industry, fall at once, when affected with even temporary mental disorder, from comfort to ruin, and whose families are hopelessly dragged down with them to poverty and want. The rich, if insanity falls upon them, are surrounded with all the care that wealth can command or sympathy suggest; the poor can apply to the parish and be received into a county asylum; but the class between the rich and the poor suffer without resources, and often long unknown. All the prolonged pains and griefs of concealed poverty beset and torture them; and no relief presents itself until they have wholly fallen into the rank of paupers.

THE BICETRE.

This large asylum is appropriated to male patients, or rather, as in the case of the Salpêtrière, a portion of the immense hospital is set apart for them, the rest being occupied by elderly or decayed tradespeople and others. About 2000 of these occupy the parts of the building first approached, and the buildings behind these contain 800 or 900 male lunatics. M. Voisin and M. Leuret are physicians to this part of the establishment; with both of whom, as well as with M. Mitivié, one of the physicians to the Salpêtrière, I had subsequent opportunities of becoming acquainted.

I was accompanied round this asylum by M. Battelle, and by M. Mallon, the director, and had afterward an opportunity of hearing from himself the exposition of the views of one of its able physicians, M. Voisin, whose singular zeal in the cause of the idiotic class of patients has caused difficulties to be overcome, which appeared at first to be insurmountable. The first part of the Bicêtre to which I was conducted was a school exclusively established for the improvement of these cases and of the epileptic, and nothing more extraordinary can well be imagined. No fewer than forty of these patients were assembled in a moderate sized school-room, receiving various lessons and performing various evolutions under the direction of a very able school-master, M. Seguin, himself a pupil of the celebrated Itard, and endowed with that enthusiasm respecting his occupation before which difficulties vanish. His pupils had been all taught to sing to music; and the little band of violins and other instruments, by which they were accompanied, was formed of the old almsmen of the hospital. But all the *idiotic* part of this remarkable class also sung without any musical accompaniment, and kept excellent time and tune. They sung several compositions, and among others a very pretty song, written for them by M. Battelle, and sung by them on entering the class-room. Both the epileptic and idiotic were taught to write, and their copy-books would have done credit to any writing-school for young persons. Numerous exercises were gone through, of a kind of military character, with perfect correctness and precision. The youngest of the class was a little idiot boy of five years old, and it was interesting to see him following the rest, and imitating their actions, holding out his right arm, left arm, both arms, marching to the right and left, at the word of command, and to the sound of a drum beaten with all the lively skill of a French drummer by another idiot, who was gratified by wearing a demi-military uniform. All these exercises were gone through by a collection of beings offering the smallest degree of intellectual promise, and usually left, in all asylums, in total indolence and apathy. Among them was one youth whose intellectual deficiency was marked in every look, gesture, and feature.

I think a more particular account of this poor boy's progress deserving of record, as an inducement to the philanthropist to enter on a new field of instruction presenting many difficulties, but yet not unproductive of results. But I must premise that to M. Voisin, one of the physicians of the Bicêtre, the honour seems chiefly if not wholly due of having attracted attention to the various characters of idiots, and their various capacities, with a view to cultivating, with precise views, even the fragmentary faculties existing in them. His work, entitled 'De l'Idiotie chez les Enfants,' abounds with remarks calculated to rescue the most infirm minds from neglect, and to encourage culture in cases before given up to despair. Fourteen years' experience has confirmed the soundness of his opinions; and they have had the sanction of MM. Ferrus, Falret, and Leuret, physicians of the highest distinction in the department of mental disorders. M. Ferrus, who is the President of the Academy of Medicine, and Inspector-General of the Lunatic Asylums of France, was, indeed, the first to occupy himself, so long ago as in 1828, with the condition of idiots at the Bicêtre, of which hospital he was the chief physician. He organized a school for them, caused them to be taught habits of order and industry, and to be instructed in reading, writing, arithmetic, and gymnastic exercises. M. Voisin's first publication on the subject appeared in 1830. The efforts of M. Falret at the Salpêtrière for the instruction of the insane, already spoken of, began in 1831 by the establishment of a school in that institution for idiotic females. Nine years later, MM. Voisin and Leuret, as physicians to the Bicêtre, organized a system of instruction and education on a greater scale. These benevolent and successful efforts deserve to be remembered, as they no doubt prepared the way for the systematic attempt since made at the Bicêtre, where M. Seguin is enabled to apply to practice principles of tuition long recognized as regards the deaf and dumb, but only beginning to be acknowledged as respects those unfortunate beings whose mental faculties are congenitally imperfect in all the various degrees classed under the term idiocy. In this application the master has to educate the muscular system and the sensorial apparatus, as well as the intellectual faculties, or rather the intellectual faculties through them, as a preliminary; doing, in fact, for them by art, by instruction, by rousing imitation, what nature does for healthier infant organizations. The healthy infant is placed in a world calculated to exercise its senses and to evoke and perfect all its muscular powers, and, to a certain extent, its intellectual faculties. The imperfect or idiotic infant is in the same world, but its senses are, to a great extent, closed to these natural influences, and its powers of muscular motion are incomplete; its intellectual faculties are not evoked by these means, and are even incapable of being fully evoked by any means whatever. The attention is vague, the memory feeble, the imagination futile, comparison is most limited, judgment most imperfect, and all the affections, sentiments, and moral qualities are disordered or perverted. The interesting question is, to what extent can careful and skilful instruction make up for these natural deficiences; and, as already done for the deaf, the dumb, and the blind, reclaim for these unfinished creatures the powers and privileges of life. The exertions of future philanthropists will answer this question. Improvement must not be looked for beyond what is strictly relative to the imperfect individual in each case; but it would seem to be true of idiots, as of the insane in general, that there is no case incapable of some amendment; that every case may be improved, or cured, up to a certain point,—a principle of great general importance in reference to treatment.

In the school for idiots and epileptics, at the Bicêtre, a careful register is kept of the psychological condition of each pupil, according to a printed form, for the examination of their instinctive, moral, intellectual, and perceptive state. I was obligingly furnished with a copy of the register relative to the subject of my immediate observations, *Charles Emile,* and also with a copy of the *résumé* or summary of his case, made by M. Voisin himself.

The age of Charles Emile is fifteen: he was admitted to the school in June, 1843. He is described as being of a nervous and sanguine temperament, and in an almost complete state of idiocy; the faculties which remain being in a state of extraordinary activity, and rendering him dangerous to himself and to others; but still idiotic in his inclinations, sentiments, perceptions, faculties of perception and understanding, and also of his senses, of which some were obtuse, and others too excitable. He was consequently unfit, to use the words of M. Voisin, "to harmonise with the world without." As regards his *inclinations,* he was signalized by a voracious, indiscriminate, gluttonous appetite, *un érotisme hideux,* and a blind and terrible instinct of destruction. He was wholly an animal. He was without attachment; overturned everything in his way, but without courage or intent; possessed no tact, intelligence, power of dissimulation, or sense of property; and was awkward to excess. His *moral sentiments* are described as *null,* except the love of approbation, and a noisy instinctive gaiety, independent of the external world. As to his *senses,* his eyes were never fixed, and seemed to act without his will; his taste was depraved; his touch obtuse; his ear recognized sounds, but was not attracted by any sound in particular; and he scarcely seemed to be possessed of the sense of smell. Devouring everything, however disgusting; brutally sensual; passionate,—breaking, tearing, and burning whatever he could lay his hands upon; and if prevented from doing so, pinching, biting, scratching, and tearing himself, until he was covered with blood. He had the particularity of being so attracted by the eyes of his brothers, sisters, and playfellows, as to make the most persevering efforts to push them out with his fingers. He walked very imperfectly, and could neither run, leap, nor exert the act of throwing; sometimes he sprang like a leopard, and his delight was to strike one sonorous body against another. When any attempt was made to associate him with the other patients he would start away with a sharp cry, and then come back to them hastily. M. Voisin's description concludes with these expressions: "All the faculties of perception in this youth are in a rudimentary state; and if I may venture so to express myself, it is incredibly difficult to draw him out of his individuality, to place him before exterior objects, and to make him take any notice of them. It would not be far from the truth to say, that for him all nature is almost completely veiled."

This description not only exemplifies M. Voisin's careful mode of observation, but shows that an example of idiocy less favorable to culture could scarcely have been presented to the instructor. This same poor idiot boy is now docile in his manners, decent in his habits, and capable, though not without some visible effort, of directing his vague senses and wandering attention, so as to have developed his memory, to have acquired a limited instruction concerning various objects, and to have become affectionately conscious of the presence of his instructors and friends. His general appearance is still that of an idiot. His countenance, his mode of walking, all that he does, declare his very limited faculties. Nature has placed limits to the exercise of his powers which no art can remove. But he is redeemed from the constant dominion of the lowest animal propensities; several of his intellectual faculties are cultivated, some have even been called into life, and his better feelings have acquired some objects and some exercise. In such a case as this we are not so much to regard what is merely accomplished for the individual. A great principle is established by it in favour of thousands of defective organizations. After witnessing the general efforts of this school of the most imbecile human beings, and hearing the particulars of Charles Emile's history, it was really affecting to see him come forward when called, and essay to sing a little solo when requested; his attempt at first not being quite successful, but amended by his attention being more roused to it. His copy-book was then shown to me, and his writing was steady, and as good as that of most youths of his station in life. The schoolmaster, who seemed to take great pleasure in the improvement of this poor fellow, then showed us how he had taught Charles to count, by means of marbles and small pieces of wood, or marks made on a board, arranged in lines, the first containing an 0, the second 00, the third 000, and so on. Charles was sometimes out in his first calculations, but then made an effort and rectified himself. He distinguished one figure from another, naming their value. Large pieces of strong card, of various shapes, were placed in succession in his hands; and he named the figure of each, as square, triangle, &c. and afterward drew their outlines with chalk on a black board, and, according to the desire of M. Seguin, drew a perpendicular, or horizontal, or oblique line; so effectually attending to what he was doing, that if any line was drawn incorrectly he rubbed it out and began anew. He also wrote several words on the board, and the name of the director of the Bicêtre, without the name being spoken to him.

This case was altogether the most interesting of those which I saw; but there was one poor idiot standing a great part of the time in a corner, to all appearance the very despair of art: even this poor creature, however, upon being noticed and brought to the table, proved capable of distinguishing the letters of the alphabet. Most of the others had received as much instruction as has been described, and could count, draw lines and figures, write, perform various exercises, and point to different parts of the body, as the head, the eyes, the arms, the feet, &c., when named to them. In all these cases, and pre-eminently in that of Charles Emile, the crowning glory of the attempt is, that whilst the senses, the muscular powers, and the intellect have received some cultivation, the habits have been improved, the propensities regulated, and some play has been given to the affections; so that a wild, ungovernable animal, calculated to excite fear, aversion, or disgust, has been transformed into the likeness and manners of a man. It is difficult to avoid falling into the language of enthusiasm on beholding such an apparent miracle; but the means of its performance are simple, demanding only that rare perseverance without which nothing good or great is ever effected; and suitable space, and local arrangements adapted to the conservation of the health and safety of the pupils; to the establishment of cleanly habits; to presenting them with objects for the exercise of their faculties of sense, motion, and intellect; and to the promotion of good feelings and a cheerful active disposition. The idiot who is capable of playing and amusing himself is already, as M. Seguin observes, somewhat improved.* I can but regret that I had not time to watch the progress of this interesting school from day to day, and to trace the growth of knowledge in the different pupils; as of the first ideas of form and colour, into writing and drawing; the development of articulation and the power of verbal expression; the extension of memory to calculation; the subsidence of gross propensities, and the springing forth and flourishing of virtuous emotions in a soil where, if even under the most favorable circumstances the blossoms and fruits are few, but for philanthropic culture all would be noxious or utterly barren.

The schools for the insane patients of the Bicêtre, who are neither idiotic nor epileptic, exceed in interest, if possible, those of the Salpêtrière. Male patients are better prepared in general than female patients to derive benefit from such instruction; they are also more attentive and, perhaps, more able to receive various instruction. I have never seen more exquisite penmanship than that of some of the male patients; the drawings of some of them were most beautiful; and I will not attempt to describe the effect of their singing, although I can never lose the impression of it. Here, too, as in the school at the Salpêtrière, the most cheering thing of all was to see the evident comfort and happiness created by the various and not fatiguing occupations of the schools; to witness the satisfaction with which the afflicted, the paralysed, the utterly incurable, exhibited the performances which they yet retained the power to accomplish. If no other end were answered by the formation of schools, they ought to be established as recreative, palliative, remedial even, in every lunatic asylum.

There is no asylum in which the good effect of occupation has been tried to a greater extent than at the Bicêtre. The exertions of M. Ferrus long since procured for this purpose the farm of St. Anne, at some distance from the asylum. Unfavorable weather and want of time contributed to preveut my visiting this farm, the extent of which is, I believe, about 150 acres. Its cultivation has realized the most sanguine expectations of the physicians as regards the bodily and mental improvement of the patients employed upon it; and, what is of far less consequence, has actually been profitable. A simple regard to the profit of occupations for the insane will always limit the application of this most important remedy; and it is, therefore,

* Hygiène et Education des Idiots. Par Edouard Seguin. Paris, 1843.

satisfactory that the farming at St. Anne has not been a source of loss. The patients in the schools, even some of the epileptic and idiotic, work when the weather permits it; and for some, who are employed nearly every day, there are evening classes. Thus every objection is removed which can be raised against the instruction of the insane, even by those who regard economy as the first consideration. Out of 800 male patients, 200 receive instruction.

Although farm-labour is not the only occupation of the patients at the Bicêtre, it is yet probable that a greater variety of occupations might be furnished. This is certainly the case in most of the English asylums. The asylum at Glasgow is, perhaps, the only British asylum in which there is a printing-press; but patients cannot be employed without having superintendents and teachers of work, and proper work-rooms; and the dread of expense is the explanation of the crowd of unemployed men in all the wards of our lunatic institutions.

The general arrangements in the wards of the Bicêtre are of the most comfortable description. The same order and cleanliness, the same industry and tranquillity, are observable as at the Salpêtrière. The visitor is not furnished with reports of the asylums in Paris, where either such reports are not published, or are as rarely presented to a stranger as at Bethlem or St. Luke's. In all our county asylums the reports and statistical registers, &c , are readily procured, and offer a kind of security for general good arrangements, as well as much useful information. I am unable, consequently, to give any diet table of the Bicêtre or of the Salpêtrière, or any details with regard to the clothing of the patients, per centage of deaths or recoveries, &c. The appearance of the patients indicates that their diet is in no respect defective; and the meat in the slaughter-house, the bread, the soup, the vegetables, and the complicated and important performances carrying on in the kitchen, were extremely satisfactory to the spectator. The soup is certainly better than that usually produced by our English cooks in such institutions. The clothing of the French patients is capable of much improvement, and there is every reason to expect that it will soon be much improved. The excuse alleged for its present state is want of ample funds. The laundry arrangements are very good, although very different from those of our asylums.

Few of the patients at the Bicêtre were violent, few in seclusion, none in restraint. Everything is done to habituate them to social life. They sleep in large dormitories, work together, dine together, read, write, and amuse themselves together, attend the services of religion together. The very refractory and the furious are not often seen in asylums conducted on the principles which prevail there; but when such cases prevent themselves they are separated from the rest; all irritations are carefully excluded, baths and other remedial means are resorted to, and no violent methods of repression sanctioned. Such at least appears to be the rule, and I saw no probable evidence of exceptions, no chains or cords, no manacles or leg-locks, no bath of surprise, no whirling chair, no boxes or osier baskets for inclosing the body and limbs of the turbulent, no patients sitting in restraint chairs as a substitute for superintendence and care. I have been told that very recently there were many, and if so, their disappearance is of good augury. It would, indeed, have been strange to find such things in an asylum made memorable by the signal services rendered to the insane by Pinel; for it was here, in basement-dungeons and dark and dreary cells shut out from the light of day, that this great physician liberated fifty-three captive lunatics with his own hand. The epoch (1792) was unfavorable to any humane enterprise, and vitiated in some degree the good effect of this; but its blessed influence has never since been wholly lost.

It is to be remembered that at that time all the rigours of treatment were sanctioned by the highest medical authorities. Very humane physicians recommended the flogging of lunatics as a part of their regular treatment. The insane, it was said, were to be subdued ; the depressing passions were the most likely to subdue them ; fear was the most powerful of the depressing passions ; and this was most surely, readily, and conveniently excited by stripes. On this conclusive reasoning was founded a decisive practice; and patients were scourged without mercy and without remorse. This horrible doctrine, and the usages founded upon it, were struck down by the courage of Pinel ; not at once resigned ; clung to, as all cruel modes of restraint yet are, but at least discountenanced for evermore.

We can never do too much honour to the memory of Pinel. Without examples to animate him, or protection or sympathy to encourage him, he proceeded alone in this great undertaking; disregarding personal risk and probable loss of reputation; and although the objects of it had well nigh forgotten the use of their senses and their limbs, benumbed by years of captivity in some cases amounting to forty, he had the great reward of seeing tranquillity succeed in the asylum to constant tumult, and even some of these patients eventually recover their reason. Apart from the great transactions on which the destinies of nations have turned, there is not, perhaps, in the annals of mankind any action more great and noble than this. Only fifty were set free by it, but it has subsequently regained or preserved freedom to thousands who would have sighed and died in prisons and caves too dreadful for description, but for PINEL.

As a proof of the rapid progress of improvement in the Bicêtre, where in November I found no restraint, or instrument of restraint, it is now not only curious but even gratifying to me to mention the following circumstances connected with this asylum, as illustrative both of the concomitant evils of the great evil of restraint, and the advantage of discontinuing it. The Bicêtre was visited by my son, and some other members of my family, accompanied by M. Battelle, three months before my own visit to Paris. At that time there were some patients fastened in chairs, of some of whom my son wrote me the following account:

"Passing to an up-stairs room, we found therein a number of beds with a restraint-chair between each, very similar to those formerly used at Hanwell. In one of these sat a poor emaciated creature with a strait-waistcoat over his chest and arms, a broad strap round his waist fastening him to his seat, and a number of linen rags and ropes round his legs; this man was talking loudly and incoherently, but with a breaking voice that showed debility and exhaustion. Being asked by M. Battelle what we would do with such a man at Hanwell, I inquired what the man would do if at large. It was stated by the attendant that he would tear his clothes; and it seemed that he had been five months in restraint, and that at night he was tied down in his bed. I was not surprised to hear it added of him that he 'never slept.'

"On the opposite side of the room sat a man more miserable looking than the other, and restrained in a similar way. I forgot to say that the other man had no shoes or stockings on: this man was in the same condition. The little clothing they had on was very dirty; this second man had not been shaved for some time. I was told that he beat the other patients; and M. Battelle asked me what I would do in *such* a case. I replied, if those two men were brought to Hanwell they would certainly be released immediately, and no doubt some means would be found to keep them from doing any harm. M. Battelle then proposed to the superintendent that this second man should be released, that I might see what he would do. I said I should attach no importance to such an experiment, unless I could remain to see efficient care and kindness substituted for restraint; that the *immediate* effect of release would probably be violence, and so on. However, it was determined to try; and the four stout attendants with us summoned about ten others before venturing to loosen the bonds. We had left the ward and were in an airing court when the patient was brought down with a strong body-guard: and in what way will it be supposed that this furious maniac was venting his rage? By talking affectionately, crying, and kissing the attendants all round. He was then put into a solitary cell, and locked up, as if such was the essence of the non-restraint system."

Recollecting these two cases, it was my intention to notice them, or inquire about them; but M. Battelle had the candour to allude to them without any inquiry being made; and I had the satisfaction to learn that the man so liberated had got well, and that the other patient had also been liberated and was improving. How far the liberation conduced in one case to recovery, and in the other to amendment, I do not pretend to determine; but the circumstances attending the liberation declare a disposition in the officers of the French asylums which is highly favorable to every kind of improvement.

Still, even my experience at Hanwell, where I have had the good fortune to act under a Committee which has uniformly supported the principle of non-restraint for five years, makes me apprehensive lest the imperfect manner in which it is carried out should discredit a principle so excellent. Non-restraint requires for its success certain expedients for the protection of clothing and property. It requires a body of well-chosen attendants; governed with justice and mercy. It requires a well-arranged building. Above all it demands compassionate and intelligent officers to direct the whole. Where any one of these conditions is wanting, the experiment will eventually fail. The real friends of non-restraint in our asylums are yet very few in number. A chief officer who enforces it may be insidiously opposed in every department, with little show of opposition. The old methods were gratifying to all who love power and dislike trouble. A new generation of officers, better educated, and an improved discipline and government of our asylums, securing proper authority to those intrusted with the care and cure of the insane, and alone responsible for it to the public under every disadvantage, will establish the great truth, that the only restraint which is universally to be depended upon is the uniformly kind and indulgent treatment of those whose malady has reduced them to the feeble and wayward condition of childhood.

[The remainder of the notices, containing an account of Charenton, of St. Yon, near Rouen, &c. will appear in our next Number.—Ed.]

